# Transcriptome analysis reveals immune and metabolic regulation effects of *Poria cocos* polysaccharides on *Bombyx mori* larvae

**DOI:** 10.3389/fimmu.2022.1014985

**Published:** 2022-10-28

**Authors:** Jiajie Liu, Wanyu Hong, Mei Li, Yang Xiao, Yunhong Yi, Yi Liu, Gongqing Wu

**Affiliations:** ^1^ School of Chemistry and Chemical Engineering, Guangdong Pharmaceutical University, Zhongshan, China; ^2^ Zhongshan Institute, University of Electronic Science and Technology of China, Zhongshan, China; ^3^ Sericultural and Agri-Food Research Institute, Guangdong Academy of Agricultural Science, Guangzhou, China; ^4^ Guangdong Cosmetics Engineering & Technology Research Center, Zhongshan, China

**Keywords:** silkworm, immunoregulation, transcriptome analysis, metabolic alterations, innate immune parameters

## Abstract

*Poria cocos* polysaccharides (PS) have been used as Chinese traditional medicine with various pharmacological effects, including antiviral, anti-oxidative, and immunomodulatory activities. Herein *Bombyx mori* silkworm was used as a model animal to evaluate the immunomodulatory effects of PS *via* detecting the changes of innate immune parameters and explore the underlying molecular mechanism of the immunoregulatory effect of PS using Illumina HiSeq Xten platform. The results presented here demonstrated that a hemocoel injection of PS significantly enhanced the cellular immunity of silkworm, including hemocyte phagocytosis, microaggregation, and spreading ability. A total of 335 differentially expressed genes (DEGs) were screened, including 214 upregulated genes and 121 downregulated genes by differential expression analysis. Gene annotation and enrichment analyses showed that many DEGs related to immune signal recognition, detoxification, proPO activation, carbohydrate metabolism, and lipid metabolism were significantly upregulated in the treatment group. The Kyoto Encyclopedia of Genes and Genomes-based Gene Set Enrichment Analysis also revealed that the more highly expressed gene sets in the PS treatment silkworm were mainly related to immune signal transduction pathways and energy metabolism. In addition, the activity of four enzymes related to immunity and energy metabolism—including phenoloxidase, glucose-6-phosphate dehydrogenase, hexokinase, and fatty acid synthetase—were all significantly increased in the larvae injected with PS. We performed qRT-PCR to examine the expression profile of immune and metabolic-related genes, which further verified the reliability of our transcriptome data and suggested that PS can regulate the immunity of silkworm by enhancing the cellular immunity and modulating the expression levels of genes related to immune responses and physiological metabolism. These findings will lay a scientific foundation for the use of PS as an immunomodulator in disease prevention in human beings or animals.

## Introduction

*Bombyx mori*, belonging to the silkworm moth family of Lepidoptera, is an oligophagous and holometabolous insect that feeds on mulberry leaves, and it is an ideal animal model with a short growth cycle. Compared with many mammal models (*e*.*g*., mouse, rat and rabbit), silkworm has many unique advantages, such as low breeding cost, fewer ethical problems, rich genetic mutation resources, clear genetic background, and suitability for large-scale breeding.

Owing to the above-mentioned advantages of silkworm as well as its similarities with mammals in basic life system, energy metabolism, and inheritance patterns, silkworm has gained increasing attention in many fields, including pharmaceutical research (1), human disease investigation (2), and toxicological research (3–5)—for example, researchers evaluated drug toxicity using silkworm as an animal model and found that the silkworm eliminated the chemicals through a common metabolic pathway with the mammals (6). By using the silkworm–baculovirus infection mode, Orihara et al. screened antiviral agents that are effective for treating humans infected with DNA viruses (7). Silkworm models are also widely used for the evaluation of anti-diabetic drugs for both type I diabetes and type II diabetes (8). Our previous report showed that silkworm possessed an immune-priming response, which was similar to the adapted immunity of vertebrates, and the specificity of the priming response was mediated by hemocyte phagocytosis (9). We further investigated the molecular mechanism of specific immune priming in silkworm using transcriptome sequencing and found that many immune-related genes, such as pattern recognition receptors, antimicrobial peptides (AMPs), and detoxification genes, were involved in the enhanced immune response (10). More and more researchers use silkworm as an alternative model of experimental animals, as it is an ideal experimental model in the fields of medicine, genetics, immunology, and toxicological research.

Fungal polysaccharide refers to a high-molecular-weight polymer with bioactive functions and connected by 10 or more monosaccharides in the form of glycosidic bonds. They are not only the main components of cells and tissues but also participate in energy storage and a variety of life activities. Fungal polysaccharides are known as “biological response modifiers” (BRMs). They have attracted much attention due to their biological activities, such as antitumor, antioxidant, anti-inflammatory, antibacterial, and immunoregulatory activities (11–15).

*Poria cocos* is a saprophytic fungus belonging to the family of Polyporaceae. Its sclerotium, called fu-ling in China, has been used as a Chinese traditional medicine for more than 2000 years. Many studies have shown that *P. cocos* polysaccharides (PS) possess excellent immunomodulatory effects—for example, a polysaccharide-rich fraction derived from *P. cocos* could improve the adaptive immune cell activation and modulate the specific immune response of mice (16). PS could exert immunomodulatory effects by activating the Ca^2+^/PKC/p38/NF-κB signaling pathway in macrophages and also serve to enhance the immune activity against lung cancer *via* TLR4/TRAF6/NF-κB signaling (17, 18). Although the past results suggested that PS displayed an immunomodulatory activity, the specific molecular mechanisms underlying how PS regulates the host immunity and metabolism system remain to be fully explored.

Insects possess effective immune systems composed of both humoral (secretion of AMPs and activation of proteinase cascades) and cellular (hemocyte phagocytosis, encapsulation and microaggregation) components (19, 20). The blood cavity of insects contains a large amount of fat body, which is the central tissue of metabolic activities such as insect growth, development, metamorphosis, and reproduction. The main functions of the fat body are storing nutrition, detoxification, and providing various biosynthetic metabolites for insect life activities, which are similar to those of the vertebrate liver. Most AMPs are also produced mainly in the fat body.

In this study, we used transcriptome sequencing technology to explore the immune signaling pathways, key metabolic pathways, and genes in the fat body of silkworm affected by PS. Many immune and metabolic-related differentially expressed genes (DEGs) were selected for further analysis by qPCR. Furthermore, we systematically analyzed the changes of innate immune parameters, such as phagocytosis, hemocyte microaggregation, and spreading of silkworm after inoculation with PS. To our knowledge, this is the first study on the molecular mechanisms of immunoregulation as exerted by PS using silkworm as an animal model. The results will lay a scientific foundation for the use of PS as an immunomodulator in disease prevention in human beings or animals.

## Materials and methods

### Silkworm rearing and survival assay

*B. mori* strain p50 was reared on mulberry leaves at 25°C with a 12-h light and 12-h dark cycle. Then, 1-day-old fifth-instar larvae were selected for the following experiments. PS (purity >90%) was purchased from Sangon Biotech (Shanghai) Co., Ltd. For the immune parameters and survival assays, four groups of larvae (50 larvae per group) were treated as follows: The first group of larvae was injected with 10 μl of phosphate-buffered saline (PBS: 8 g NaCl, 0.2 g KCl, 1.44 g Na_2_HPO_4_, and 0.24 g KH_2_PO_4_ in 1,000 ml distilled water, pH 7.2) as control. The other three groups were injected with 10 μl of PBS containing 0.1, 0.2, and 0.4 µg PS, respectively. The preliminary experiments showed that PS had no lethal effect on the silkworm larvae in the range of the concentrations selected. At 24 h after injection, 30 larvae of each group were selected and injected with the lethal doses of (1 × 10^5^ cells/larva) *Bacillus thuringiensis* (*Bt*) for the survival assay. The hemolymph was collected from the remaining larvae of each group for microaggregate counts, phagocytosis, and hemocyte spreading assays. For all the treatments, three independent trials were performed.

### Hemocyte spreading and microaggregate count assays

In detail, 50 µl of hemolymph was diluted into five volumes of ice-cold anticoagulant solution (93 mM NaCl, 100 mM glucose, 30 mM trisodium citrate, 26 mM citric acid, 10 mM Na_2_EDTA, and a few crystals of phenylthiourea, pH 4.6.) and centrifuged at 800 g for 5 min. The pellet was resuspended in 200 µl of Grace’s tissue culture medium (GIM). After incubation at 28°C for 1 h in a 24-well plate, the hemocyte spreading behavior was observed under a phase-contrast microscope by counting the hemocyte-exhibiting cytoplasmic extension along with pseudopodial growth. Hemocyte spreading was expressed as the percentage of spreading plasmatocytes to the total number of plasmatocytes.

Moreover, 20 µl of hemolymph was diluted into 80 µl PBS with a few phenylthioureas and added to a Neubauer hemocytometer immediately. The number of microaggregates was determined directly by phase-contrast optics. Only hemocyte clusters containing five cells or more were considered as microaggregates. The number of microaggregates was normalized to microaggregates/µl hemolymph.

### *In vitro* phagocytosis assay

To prepare fluorescein isothiocyanate (FITC-labeled bacteria, heat-killed *Escherichia coli* was resuspended in carbonate buffer (0.2 M Na_2_CO_3_ and 0.2 M NaHCO_3_, pH 9.4) containing FITC (0.1 mg/ml) and then incubated in a rotary mixer (200 rpm) for 30 min in the dark at 28°C. After removing the free FITC, the suspension was diluted with GIM to a concentration of 10^9^ cells per milliliter. An *in vitro* phagocytosis assay was conducted as described in our previous study (9). The phagocytosed FITC-labeled *E. coli* was detected by fluorescence microscopy, and the average fluorescence intensity of each phagocytic hemocyte was quantified by Image J software.

### Enzyme activity assay

Two groups (45 larvae per group) of *B. mori* larvae were selected for the enzyme activity assay. One group was injected with PS (0.2 µg/larva) solution, and the other was injected with PBS and served as the control. At 24 h after injection, the fat body of each larva was isolated and ground with liquid nitrogen using a mortar. Subsequently, 1 ml of Tris-HCL (10 mmol/L, pH 7.0) was added, and the mixture was centrifuged at 10,000 rev/min for 5 min at 4°C. The supernatant was used for enzyme activity assay.

For the PO activity assay, 1.4 ml of PBS was added to 1.50 ml of L-DOPA (0.05 mol/L) and preincubated at 30°C for 30 min. Then, 0.1 ml of enzyme solution was added, and phenoloxidase (PO) activity was measured using a spectrophotometer at 490-nm wavelength. One unit of PO was described as 0.01 absorbance increase at 490 nm per minute. Glucose-6-phosphate dehydrogenase (G6PD), hexokinase (HK), and fatty acid synthetase (FAS) activities were determined using corresponding detection kits (Jiancheng, China). Total protein concentration was determined using the Bradford protein assay, with bovine serum albumin as the standard (Sigma-Aldrich, USA). Each milligram of protein that oxidizes 1 µmol of NADPH per minute was expressed as one unit of FAS activity. One unit of G6PD and HK activities were defined as each milligram of tissue protein that generates 1 nmol of NADPH per minute. Three biological replications were used to determine the average of enzyme activity in all experiments.

### Library preparation and sequencing

A total of 60 *B. mori* larvae were selected and divided into two groups—one group was injected with 10 μl of PBS containing 0.2 µg PS, and the other group was injected with PBS only as a control. All the larvae were reared on mulberry leaves. After having been fed for 24 h, the larvae were anesthetized on ice, and fat bodies were collected and frozen in liquid nitrogen quickly. Total RNA was extracted from the fat body using TRIzol^®^ Reagent according to the manufacturer’s instructions (Invitrogen), and genomic DNA was removed using DNase I (TaKara). Then, RNA quality was determined by 2100 Bioanalyser (Agilent). Only high-quality RNA sample was used to construct a sequencing library.

The RNA-seq transcriptome library was constructed according to the instructions of TruSeq™ RNA Sample Preparation Kit (Illumina, San Diego, CA, USA). Firstly, poly-A tail mRNA was enriched from 1 µg of total RNA by magnetic beads with oligo (DT) and then fragmented by a fragmentation buffer. Secondly, double-stranded cDNA was synthesized using a SuperScript double-stranded cDNA synthesis kit (Invitrogen, CA, USA) with random hexamer primers. Then, the synthesized cDNA was subjected to end-repair, phosphorylation, and “A” base addition according to Illumina’s library construction protocol. The libraries were size-selected for cDNA target fragments of 200–300 bp on 2% Low Range Ultra Agarose, followed by PCR amplification using Phusion DNA polymerase (NEB) for 15 PCR cycles. After having been quantified by TBS380, the paired-end RNA-seq sequencing library was sequenced with the Illumina HiSeq xten sequencer (2 × 150-bp read length).

### Differential expression analysis and functional enrichment

To identify differentially expressed genes (DEGs) between the PS treatment and the control group, the expression level of each transcript was calculated according to the fragments per kilobase of exon per million mapped reads (FPKM) method. RSEM was used to quantify gene abundances. The differential expression level was analyzed by R statistical package software EdgeR. Gene Ontology (GO) and Kyoto Encyclopedia of Genes and Genomes (KEGG) functional enrichment analyses were carried out by Goatools (https://github.com/tanghaibao/Goatools) and KOBAS (http://kobas.cbi.pku.edu.cn/home.do) (21).

### Gene set enrichment analysis

All differentially expressed genes, whether significant or not, were used for Gene Set Enrichment Analysis (GSEA) analysis (http://software.broadinstitute.org/gsea/index.jsp). GSEA was carried out with default algorithm as 1,000 permutations, with a maximum term size of 500 and a minimum term size of 15. The enrichment score (ES) for each gene set is calculated using the entire ranked list, which reflects how the genes for each set are distributed in the ranked list. The ES and normalized ES (NES) were determined for each gene set. The significant enrichment of a gene set was assigned based on nominal *p*-value <0.05 and false discovery rate <0.25.

### Quantitative real-time PCR

All primers of the candidate gene were designed using Primer 5.0 software, and the sequences are provided in **Supplementary Table S1**. One microgram of total RNA was used for the cDNA synthesis using HiScript Q RT SuperMix (Vazyme). qRT-PCR analysis was performed using ChamQ SYBR Color qPCR Master Mix (Vazyme) and carried out on a ABI7300 apparatus (Applied Biosystems, UK) with the following program: initial denaturation at 95°C for 5 min, followed by 40 cycles of 5 s at 95°C, 30 s at 58°C, and 40 s at 72°C. Each sample was run in triplicate, and the average threshold cycle (Ct) was calculated. The glyceraldehyde-3-phosphate dehydrogenase (*GAPDH*) gene was used to normalize the expression levels, and the relative gene expression levels of the target genes were calculated using the 2^-ΔΔCT^ method (22).

## Results

### The changes of innate immune parameters and resistance to *Bt* pathogens of *B. mori* larvae inoculated with PS

**Figure 1:**
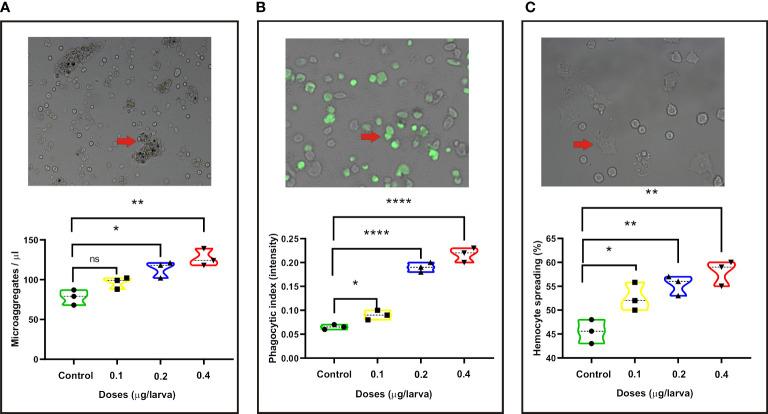
Changes in the following innate immune parameters of silkworm at 24 h after injection with different doses of *Poria cocos* polysaccharides (PS). **(A)** Microaggregate numbers. **(B)** Phagocytic index. **(C)** Hemocyte spreading. The average values calculated from three independent experiments and the statistical differences between the treatment and the control groups are displayed with an asterisk (*). *P < 0.05, **P < 0.01, ****P ≤ 0.0001.

In order to assay the immune regulation of PS on the silkworm, we inoculated the larvae with different doses of PS and examined the changes of innate immune parameters. We found that there was no significant change of microaggregate number in the hemolymph of larvae injected with a low dose (0.1 μg/larva) of PS. However, compared with the control, the number of microaggregates has significantly increased in the middle (0.2 μg/larva) and high (0.4 μg/larva) doses of PS treatment group (**Figure 1A**). Meanwhile, a stronger activation effect of phagocytic and hemocyte spreading abilities could be observed in the low, middle, and high doses of PS treatment groups because they showed significantly higher phagocytic index and hemocyte spreading ability (**Figures 1B, C**).

**Figure 2:**
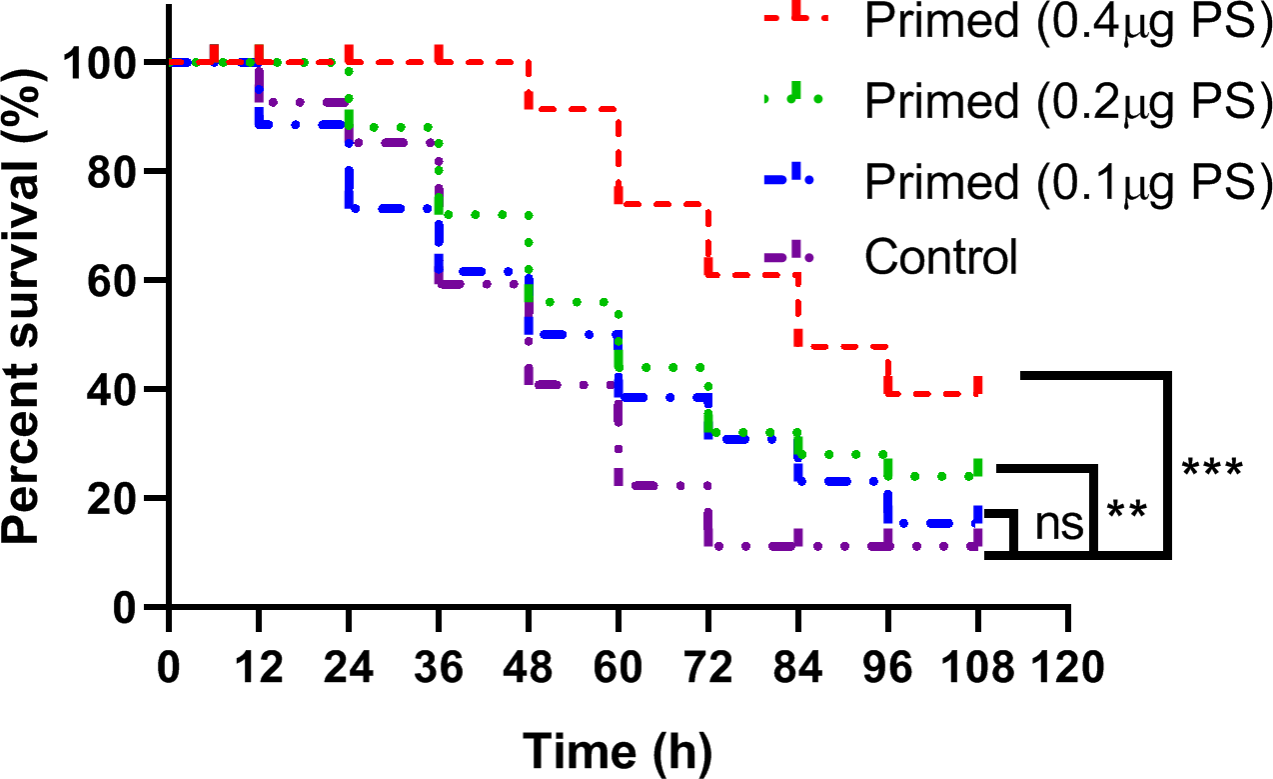
Kaplan–Meier curve analysis of *Bombyx mori* larvae immune-primed with different doses of *Poria cocos* polysaccharides followed by infection with a lethal dose of *Bacillus thuringiensis* at 24 h post-priming. ** , *** and ns indicate significance at P≤0.01, P≤0.001 and non-significant, respectively.

The larvae primed with high (log-rank Mantel–Cox test, *χ* ^2^: 14.94, *p* < 0.001) and middle (log-rank Mantel–Cox test, *χ* ^2^: 13.77, *p* < 0.01) doses of PS showed significantly higher survival rates than that of the PBS control when they were infected with the lethal dose of *Bt* at 24 h after priming, while there was no significant difference between the low dose of PS priming group and the control group (**Figure 2**).

### Quality assessment of transcriptome data

In total, 145,514,334 and 150,376,202 raw reads were gained, respectively, from the PBS (control_1, control_2, and control_3) and PS-treated (PS_1, PS_2, and PS_3) samples using an Illumina HiSeq NovaSeq 6000 platform. After quality filtering and trimming, 45,850,194 to 52,154,744 reads were obtained for six samples with high Q20 and Q30 quality scores, resulting in 87.75–89.73% of the clean reads that were mapped to the *B. mori* genome (**Supplementary Table S2**). From these six libraries, 79.54, 77.52, 76.26, 77.15, 76.71, and 78.47% reads were mapped to the CDS regions of the reference genome (**Supplementary Figure S1A**), and there was no difference in the gene expression levels among all samples (**Supplementary Figure S1B**). All reads were distributed to varying degrees on all chromosomes. Among them, the most distributed chromosome is BMSK_chr25 (**Supplementary Figure S1C**). Therefore, the data obtained from this sequencing were reliable and could be used for subsequent analysis. The original data has been uploaded to NCBI and can be found under accession numbers SRR18297772, SRR18297773, SRR18297774, SRR18297778, SRR18297779, and SRR18297780.

### Identification and functional annotation analysis of DEGs

**Figure 3:**
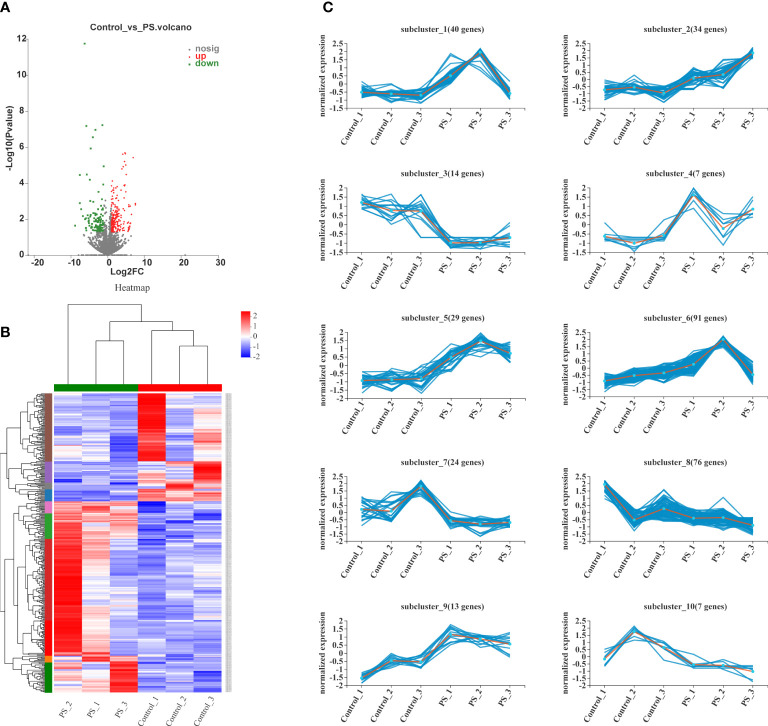
Analysis of differentially expressed genes (DEGs). **(A)** Volcano plots of the DEGs in control *vs*. polysaccharides. The red, blue, and gray dots indicate significantly upregulated, significantly downregulated, and non-significantly expressed genes, respectively. **(B)** Heat map of DEG expression profiles in all samples. The colors ranging from blue to red indicate the genes expressed from low to high levels. **(C)** Ten different expression patterns analyzed of 335 DEGs.

The volcano plots showed that 214 genes were upregulated and that 121 genes were downregulated between the control and PS-treated groups (**Figure 3A**). All DEGs were presented as FPKM hierarchical clustering heat map, through which we can observe the gene expression patterns between the controls and the PS treatment group (**Figure 3B**). To obtain more insights into the dynamic changes of DEGs, we performed a cluster analysis of 335 DEGs according to their normalized expression levels. All DEGs were grouped into 10 major clusters. DEGs that belonged to subcluster_1 (40 genes), subcluster_2 (34 genes), subcluster_4 (seven genes), subcluster_5 (29 genes), subcluster_6 (91 genes), and subcluster_9 (13 genes) were significantly induced at 24 h when the *B. mori* larvae were injected with PS. In contrast, DEGs that belonged to subcluster_3 (14 genes), subcluster_7 (24 genes), subcluster_8 (76 genes), and subcluster_10 (7 genes) showed low expression levels in the treatment group (**Figure 3C**).

**Figure 4:**
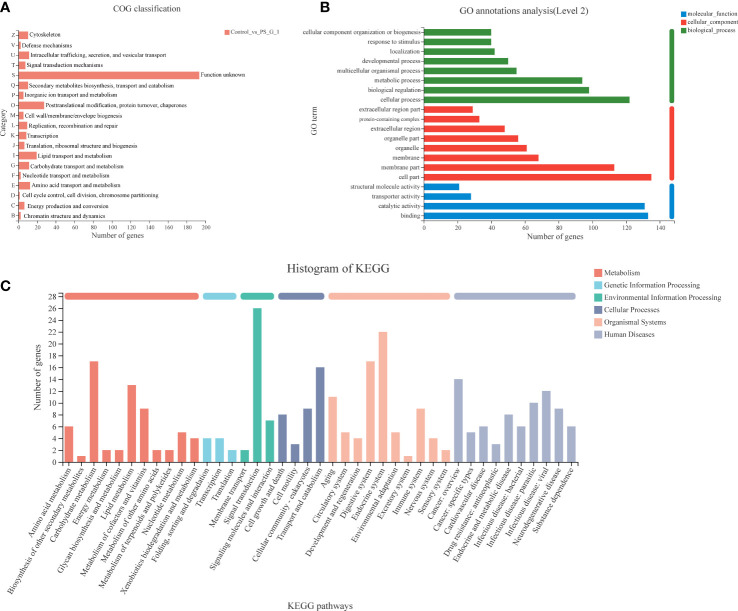
Functional classification of differentially expressed genes (DEGs) between the control and *Poria cocos* polysaccharides treatment groups. **(A)** COG, **(B)** GO, and **(C)** Kyoto Encyclopedia of Genes and Genomes annotation of DEGs.

The functions of DEGs were classified by using the COG, GO, and KEGG databases. In total, 335 DEGs were divided into 19 COG classification categories. Among the 19 COG classifications, “function unknown” represents the largest group, and “posttranslational modification, protein turnover, chaperones”, “lipid transport and metabolism”, and “amino acid transport and metabolism” are next to it (**Figure 4A**). In terms of GO annotation, all DEGs were annotated to 20 sub-categories and classified into three GO terms: biological processes, cellular components, and molecular function. The top seven subcategories were cell part (135, 40.29%), binding (133, 39.70%), catalytic activity (131, 39.10%), cellular process (122, 36.42%), membrane part (122, 33.73%), biological regulation (98, 29.25%), and metabolic process (94, 28.06%). In addition, many DEGs were annotated to respond to stimulus (40, 11.94%) and immune system process (7, 2.09%) (**Figure 4B**).

We also annotated all the DEGs based on KEGG database. A total of 335 DEGs were clustered in 41 KEGG pathways (level 2), which belonged to six categories, including metabolism, genetic information processing, environmental information processing, cellular processes, organismal systems, and human diseases. Among the 41 KEGG pathways, signal transduction (26 DEGs) was attributed with the highest number of DEGs, and the next one was the endocrine system (22 DEGs). Notably, we found that many DEGs were clustered on carbohydrate metabolism (including galactose metabolism: map00052, fructose and mannose metabolism: map00051, pentose and glucuronate interconversions: map00040, TCA cycle: map00020, pyruvate metabolism: map00620, glycolysis/gluconeogenesis: map00010, *etc.*), lipid metabolism (including fatty acid biosynthesis: map00061, glycerolipid metabolism: map00561, steroid hormone biosynthesis: map00140, *etc.*), and immune system (including hematopoietic cell lineage: map04640, Toll and Imd signaling pathway: map04624, Fc gamma R-mediated phagocytosis: map04666, NOD-like receptor signaling pathway: map04621, *etc.*) (**Figure 4C**).

### Functional enrichment analysis of DEGs

**Figure 5:**
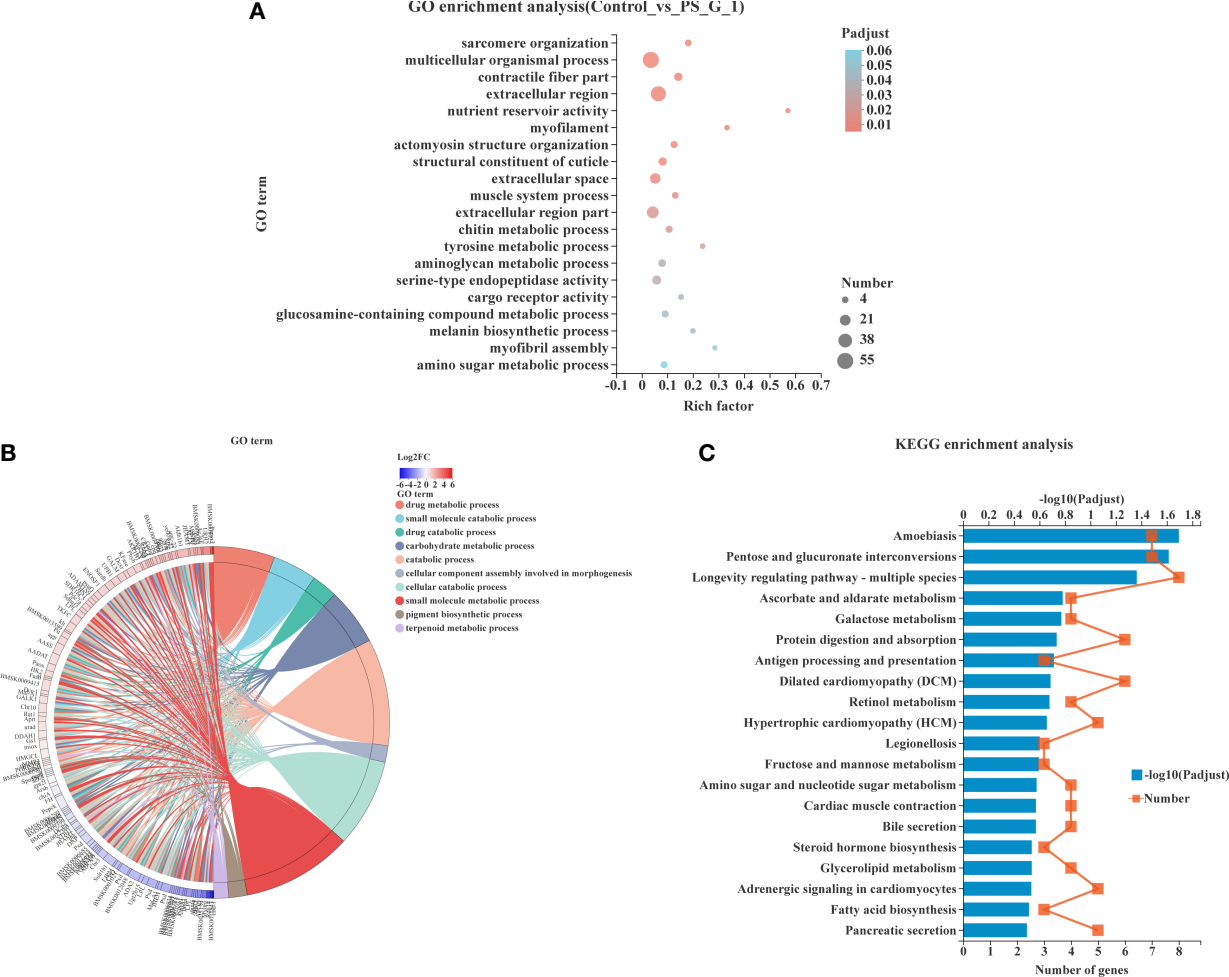
Functional enrichment analysis of differentially expressed genes (DEGs). **(A)** Gene Ontology (GO) enrichment of DEGs in silkworm exposed to *Poria cocos* polysaccharides (PS). **(B)** Chord plot indicating the relationship of the most involved genes and the top 10 terms of GO. **(C)** Kyoto Encyclopedia of Genes and Genomes pathway enrichment analysis of DEGs in silkworm exposed to PS.

The top 20 enrichment GO terms are shown in **Figure 5A**. Among the DEGs, the most enriched GO terms were multicellular organismal process (GO: 0032501), extracellular region (GO: 0005576), extracellular region part (GO: 0044421), extracellular space (GO: 0005615), and serine-type endopeptidase activity (GO: 0004252). Moreover, we performed a chord plot to analyze the top 10 enrichment GO terms, and the results showed that most of the GO terms with high enrichment were associated with metabolism, such as carbohydrate metabolic process (GO: 0005975), drug metabolic process (GO: 0017144), small molecule metabolic process (GO: 0044281), and terpenoid metabolic process (GO: 0006721) (**Figure 5B**).

The KEGG pathway enrichment analysis was also conducted to explore the possible functions of all DEGs. The top 20 enriched KEGG pathways are shown in **Figure 5C**. We found that amoebiasis (map05146), pentose and glucuronate interconversions (map00040), and longevity-regulating pathway—multiple species (map04213) were significantly enriched pathways with the largest number of DEGs being enriched.

### Gene sets involved in immune and metabolic pathways were analyzed by GSEA

We performed GSEA using the KEGG-based list to enrich the gene sets, and the GSEA enrichment plots of representative pathways are presented in **Supplementary Figure S2**. The ES, NES, *p*-value, adjusted *p*-value and leading edges are detailed in **Table 1**. The results revealed that many more highly expressed gene sets in the PS-treated group were involved in immune responses and energy metabolism. Among these gene sets, three gene sets related to immune responses, including mitogen-activated protein kinase (MAPK) signaling pathway, PI3K-Akt signaling pathway, and peroxisome, were identified as significantly enriched.

**Table 1:** List of gene set enrichment analysis statistics.

Gene set name	Description	Size	Enrichment score	Normalized enrichment score	*p*-value	*p*-adjusted	Rank at MAX	Leading edge
MAP04010	MAPK signaling pathway	221	-0.45794883	-1.571639	0	0.033788763	5,509	70
MAP04151	PI3K-Akt signaling pathway	199	-0.3764729	-1.2884775	0.03624009	0.22450094	5,633	60
MAP04146	Peroxisome	135	-0.45318866	-1.4748417	0.004705882	0.09840556	3,649	38
MAP00061	Fatty acid biosynthesis	50	-0.67814153	-1.9350237	0	0.002337374	3,443	22
MAP00010	Glycolysis/gluconeogenesis	64	-0.520225	-1.5546439	0.00656168	0.073210806	5,909	41
MAP00052	Galactose metabolism	52	-0.5516641	-1.5925494	0.004054054	0.078378215	5,270	30
MAP00020	Citrate cycle (TCA cycle)	61	-0.48012087	-1.4233419	0.037333332	0.090234995	5,204	18
MAP00051	Fructose and mannose metabolism	38	-0.5182768	-1.4233801	0.03862661	0.09492719	4,683	26
MAP00561	Glycerolipid metabolism	81	-0.47307494	-1.4593039	0.007853403	0.09939831	4,052	28

On the other hand, pathways related to glucose and lipid metabolisms, such as fatty acid biosynthesis, glycolysis/gluconeogenesis, galactose metabolism, citrate cycle (TCA cycle), fructose and mannose metabolism, and glycerolipid metabolism, were also significantly enriched and showed significant upregulation of gene sets in the PS-treated group. The results presented here showed that many of the pathways related to the immune system and energy metabolism detected by GSEA-based KEGG were overlapped with that based on DEG.

### Analysis of DEGs related to immune regulation and energy metabolism

As shown in **Table 2**, a big number of genes involved in innate immunity were significantly upregulated in the fat body of silkworm after treatment with PS, such as pattern recognition receptor genes, genes involved in prophenoloxidase-activating system, AMPs genes, and transcription factors genes. Moreover, the expression levels of genes involved in detoxification, including *Hsp68*, *CYP9E2*, *CYP6B2*, *Cyp4g15*, *sod1*, and *Csp*, were also significantly upregulated.

**Table 2:** Differentially expressed genes related to immunity, detoxification, and metabolic processes.

(blank)	Gene_ID	Gene name	Gene description	Log_2_ FC (PS/control)	*P*-value
Immune pattern recognition receptors and effectors	BMSK0006783	Toll	PREDICTED: *Bombyx mori* protein toll-like	4.3244	0.0311
	BMSK0009350	PGRP-LB	Peptidoglycan-recognition protein LB-like	-1.3431	0.0029
	BMSK0010436	Dop2R	Dopamine D2-like receptor (*Amyelois transitella*)	1.1032	0.0100
	BMSK0013513	Scarb1	Scavenger receptor class B member 1 (*Amyelois transitella*)	1.2428	0.0113
	BMSK0003099	Scarb1	Scavenger receptor class B member 1-like isoform X1	6.4726	0.0053
	BMSK0013591	SCARB2	Scavenger receptor class B member 1	2.0526	0.0276
	BMSK0013868	RyR	Ryanodine receptor-like	1.3997	0.0150
	BMSK0011182	EbpIII	Chemosensory protein isoform X1	3.2564	0.0402
	BMSK0011155	Csp1	Chemosensory protein 1	4.2132	0.0027
	BMSK0009061	ApoLp-III	Apolipophorin-III precursor	1.1019	0.0043
	BMSK0016029	Myb	Myb transcription factor	6.8827	0.0000
	BMSK0009046	GATA6	Transcription factor GATA-6 isoform X1	-1.9369	0.0143
	BMSK0009351	Sgsm1	Small G protein signaling modulator 2-like isoform X4 (*Papilio polytes*)	2.1529	0.0283
	BMSK0009367	sqh	Myosin light polypeptide 9 isoform X1	-1.0104	0.0004
	BMSK0000290	Pxn	Peroxidasin isoform X2	-7.5433	0.0005
	BMSK0007325	Lyst	Lysosomal-trafficking regulator	5.1999	0.0308
	BMSK0004847	Nurf-38	Inorganic pyrophosphatase	6.5344	0.0231
	BMSK0000559	Lyz1	Putative lysozyme	2.5224	0.0026
proPO cascade	BMSK0015957	PPAE	Prophenoloxidase-activating enzyme precursor	3.0372	0.0048
	BMSK0009085	proPO	Pro-phenoloxidase (*Bombyx mandarina*)	2.0290	0.0022
	BMSK0009190	MASP1	Serine protease gd-like	2.5316	0.0062
	BMSK0009192	lint	Serine protease gd-like	1.5159	0.0350
	BMSK0003813	Spn42Dd	Serine protease inhibitor 11 precursor	1.6572	0.0257
	BMSK0015949	SPE	Serine protease easter-like	1.4151	0.0063
	BMSK0009191	SPS-like	Serine proteinase stubble-like (*Amyelois transitella*)	2.6638	0.0072
	BMSK0012818	Serpinb1a	Serine protease inhibitor 28 isoform X2	-1.2253	0.0001
	BMSK0013357	KAZAL	Kazal-type proteinase inhibitor precursor	1.0561	0.0016
Detoxification	BMSK0011217	l(2)efl	Heat shock protein 1	1.3640	0.0433
	BMSK0009624	CYP9E2	Cytochrome P450 CYP6AE7	3.6626	0.0002
	BMSK0009632	CYP6B2	Cytochrome P450	2.9444	0.0009
	BMSK0009630	CYP6B2	Cytochrome P450 6ae2	2.6962	0.0022
	BMSK0007715	Cyp4g15	Cytochrome P450 CYP4G25	1.5695	0.0221
Peroxisome	BMSK0006862		Fatty-acyl reductase isoform X1	1.0838	0.0305
	BMSK0010527	AO2	Aldehyde oxidase 1	4.4765	0.0141
	BMSK0013700	wat	Fatty acyl-CoA reductase 1-like, partial	1.3712	0.0289
	BMSK0004505	sod1	Superoxide dismutase (Cu–Zn)	-2.2816	0.0115
	BMSK0015765		Probable phytanoyl-CoA dioxygenase	2.4666	0.0084
MAPK signaling pathway	BMSK0015669	Hsp70	Heat shock protein 70	1.9510	0.0042
	BMSK0012507	Hsp68	Heat shock protein 68	2.2055	0.0002
	BMSK0015670	Hsp70	Heat shock protein 70	2.1775	0.0305
	BMSK0000286	BBXB5	Insulin-like peptide isoform X1	1.3670	0.0492
	BMSK0015495	Ca-alpha1D	Muscle calcium channel subunit alpha-1-like Isoform X3	1.2754	0.0305
	BMSK0007881		Uncharacterized protein LOC101735793 isoform X2	1.8732	0.0117
	BMSK0001980	COL4A5	Collagen alpha-2(IV) chain	1.5978	0.0002
Carbohydrate metabolism	BMSK0012360	pik3c3	phosphatidylinositol 3-kinase catalytic subunit type 3	1.9830	0.0193
	BMSK0012645	CRYL1	XP 004931941.1 lambda-crystallin isoform X1	1.1304	0.0122
	BMSK0013964	ACLY	XP 021208590.1 ATP-citrate synthase-like, partial	1.6186	0.0003
	BMSK0000999	akr2e	Aldo-keto reductase AKR2E4-like	1.3949	0.0146
	BMSK0009262	akr2e	Aldo-keto reductase AKR2E4 isoform X1	2.6009	0.0058
	BMSK0005884	ugt-50	UDP-glycosyltransferase UGT48C1 precursor	-1.8416	0.0212
	BMSK0012235		Sucrase-isomaltase, intestinal-like (*Papilio polytes*)	-1.2959	0.0168
	BMSK0006910	UGT2A3	UDP-glycosyltransferase UGT46A2 precursor	5.7605	0.0382
	BMSK0001042	akr2e	Aldo-keto reductase AKR2E4-like	1.4045	0.0012
	BMSK0012645	CRYL1	Lambda-crystallin isoform X1	1.1304	0.0122
	BMSK0006975	Aldh1b1	Aldehyde dehydrogenase X, mitochondrial	1.4638	0.0415
	BMSK0009778	Chit1	Glycosyl hydrolases family 18	2.8129	0.0264
	BMSK0002044	chs-2	Chitin synthase	-2.6775	0.0392
	BMSK0002046	chs-2	Chitin synthase A isoform X3	2.2131	0.0037
	BMSK0004802	Ca7	Carbonic anhydrase 7 (*Plutella xylostella*)	1.3959	0.0002
	BMSK0016012	EGT	UDP-glycosyltransferase UGT33D8	1.1158	0.0441
Lipid metabolism	BMSK0003579	ACD	Acyl-CoA desaturase-like	-5.4910	0.0060
	BMSK0014844	Elovl4	Elongation of very-long-chain fatty acid protein AAEL008004-like (Amyelois transitella)	1.0341	0.0001
	BMSK0016012	EGT	UDP-glycosyltransferase UGT33D8	1.1158	0.0441
	BMSK0004214	LIPM	Lipase 1 (Papilio xuthus)	-1.5294	0.0008
	BMSK0009521	FAS	Fatty acid synthase	1.0406	0.0008
	BMSK0009527	TE-domain	Thioesterase domain	4.2394	0.0000
	BMSK0009523	pksM	Beta-ketoacyl synthase, N-terminal domain	1.5407	0.0053
Amino acid metabolism	BMSK0007388	Gnmt	Glycine N-methyltransferase	1.1500	0.0254
	BMSK0011964	Chsy1	Chondroitin sulfate synthase 1	5.3998	0.0161
	BMSK0016048	hykk	XP 012544286.1 hydroxylysine kinase	-2.6431	0.0027
	BMSK0004276	HPD	4-Hydroxyphenylpyruvate dioxygenase	5.0512	0.0000
	BMSK0004352	DNMT1	D cytosine-5 methyltransferase isoform X1	5.9968	0.0268
	BMSK0001189	FAH	Fumarylacetoacetase	1.4415	0.0356

In addition, we also analyzed the DEGs implicated in energy metabolism. Significant upregulation occurred in many genes encoding enzymes involved in carbohydrate metabolism, lipid metabolism, and amino acid metabolism.

### Expression patterns of immune-related genes and enzyme activities in the fat body of silkworm after PS injection

**Figure 6:**
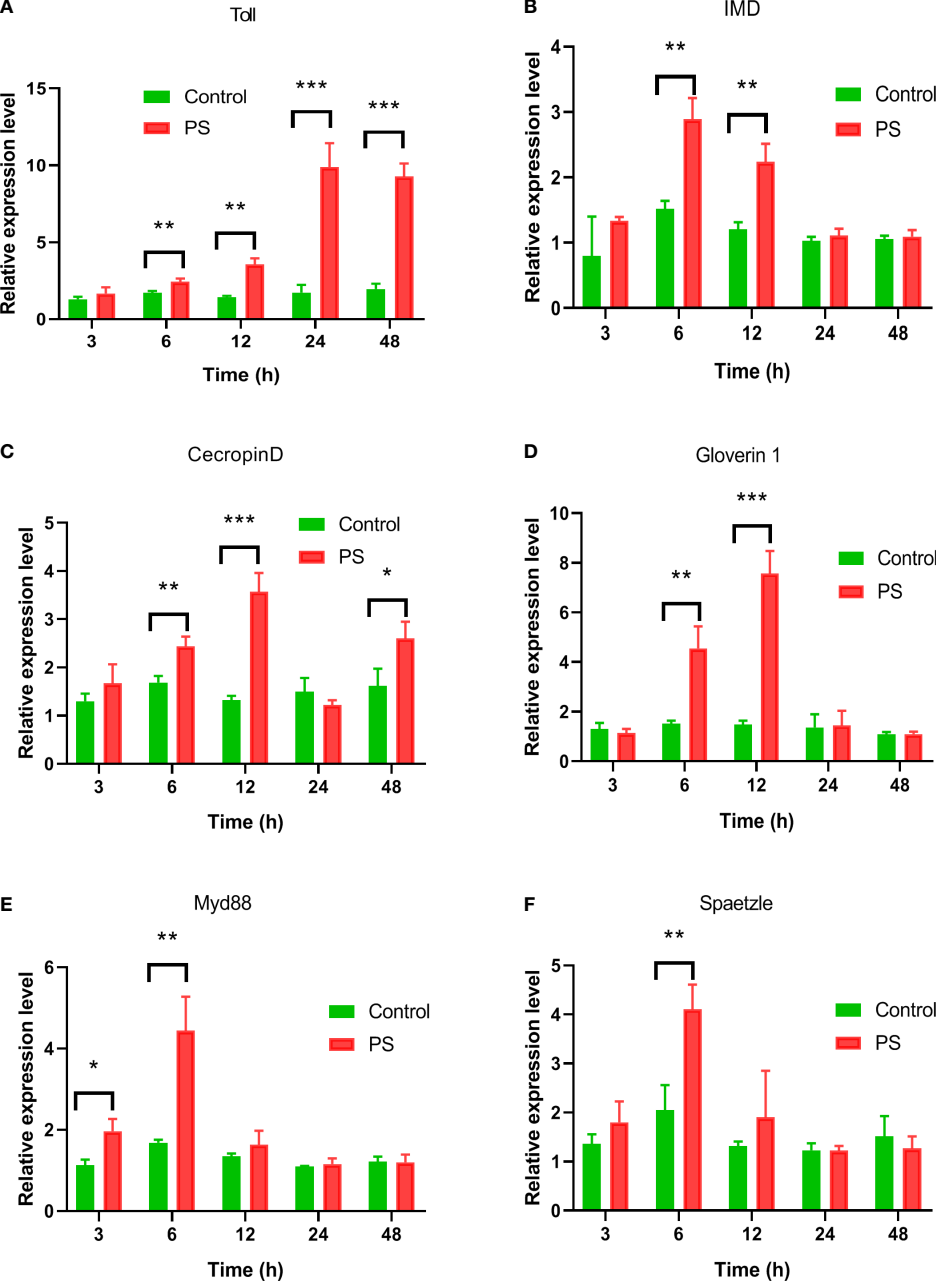
The expression profiles of genes involved in IMD, or Toll pathway induced by *Poria cocos* polysaccharides (PS). After the hemocoel injection of *Bombyx mori* larvae with PS (0.2 μg/larva), the mRNA levels of **(A)** Toll, **(B)** Imd, **(C)** Ceceropin D, **(D)** Gloverin1, **(E)** Myd88, and **(F)** spaetzle were detected by qRT-PCR. Two-way ANOVA/Tukey was conducted for statistical analysis. Asterisks indicate the levels of significant differences (*P < 0.05, **P < 0.01, ***P < 0.001).

The expression levels of genes involved in Imd and Toll signaling pathways including Toll and Imd receptors, AMP genes, *Myd88*, and *spaetzle* were detected in the fat body of silkworm after PS inoculation. Upon treatment with PS, *Toll* expression increased at 6 h and reached the highest level at 24 h, and this was significantly higher than that in the control group (*p* < 0.01); the difference lasted until 48 h (**Figure 6A**). The *Imd* expression reached the highest level at 6 h (*p* < 0.01) and subsequently decreased from 12 to 48 h (**Figure 6B**). *Gloverin1* had a similar expression pattern with *Imd*, while it reached a peak at 12 h post-injection (**Figure 6C**). The level of the *Ceceropin D* transcript increased from 6 to 12 h after PS injection and decreased dramatically to a minimum value at 24 h, while it returned to a higher level at 48 h (**Figure 6D**). Both the *Myd88* and *spaetzle* transcripts reached their peaks at 12 h (*p* < 0.01) after PS injection (**Figures 6E, F**).

**Figure 7:**
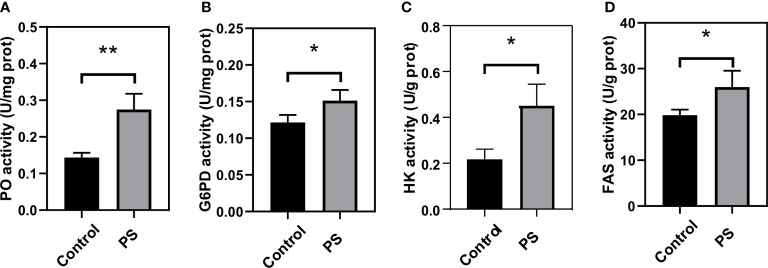
Changes of key enzymes involved in immunity and energy metabolism in the fat body of *Bombyx mori* larvae at 24 h after injecting with *Poria cocos* polysaccharides (PS). **(A)** phenoloxidase, **(B)** glucose-6-phosphate dehydrogenase, **(C)** hexokinase, and **(D)** fatty acid synthetase activities. The data was presented as mean ± SD for three independent experiments (*P < 0.05, **P < 0.01).

The activity of four enzymes related to immunity and energy metabolism was also assayed. We found that PS inoculation could significantly enhance the PO (**Figure 7A**), G6PD (**Figure 7B**), HK (**Figure 7C**), and FAS (**Figure 7D**) activities. They reached levels of up to 0.275 (U/mg protein), 0.151 (U/mg protein), 0.453 (U/g protein), and 25.97 (U/g protein) at 24 h, respectively, and were significantly higher than those of the control group.

### Verification of DEGs by qRT-PCR

**Figure 8:**
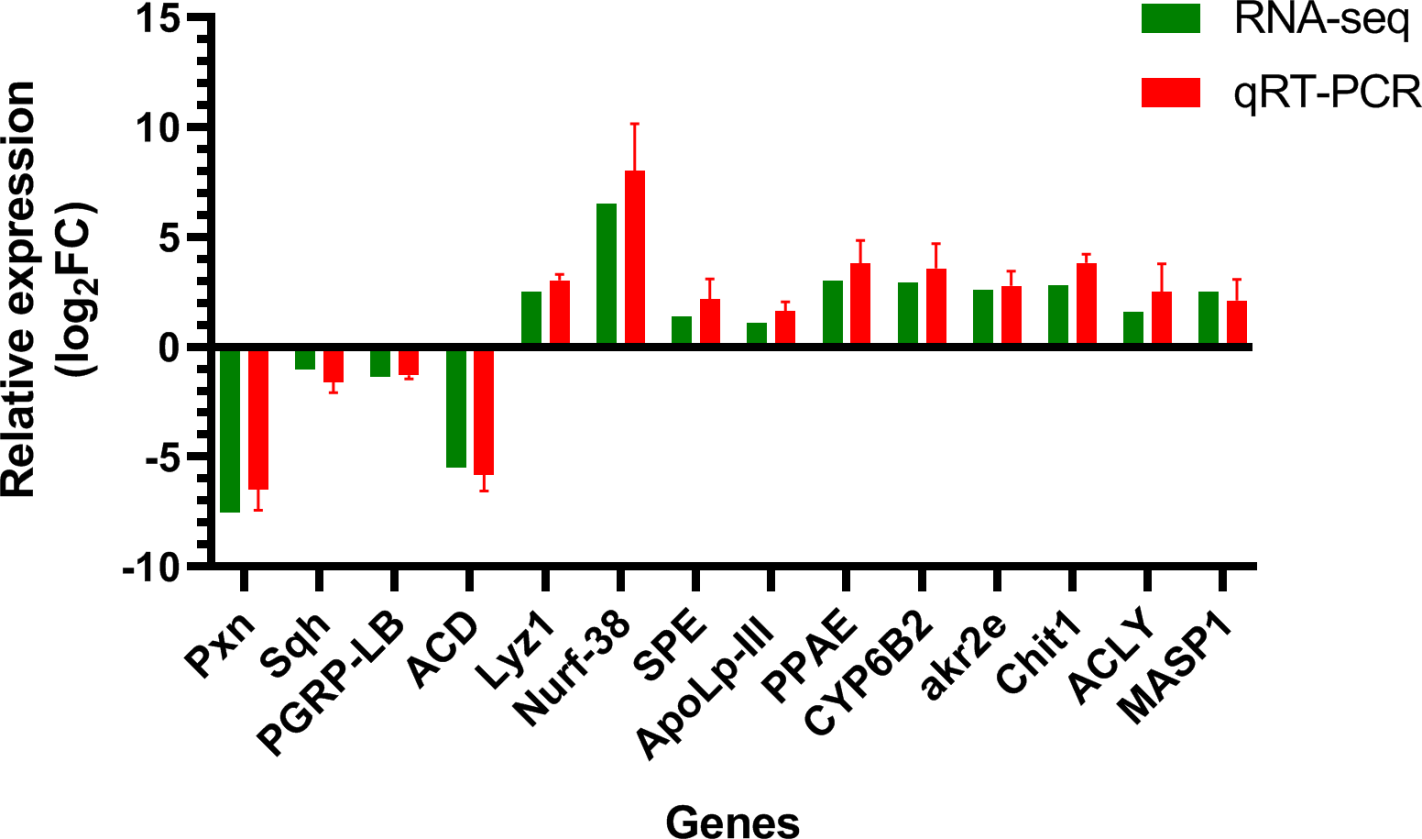
Validation results of RNA-seq profiles by real-time PCR. The y-axis indicates the value of relative expression level (2^-ΔΔCt^) by qRT-PCR and log_2_ ratio of PS/control RNA-seq. Pxn, peroxidasin isoform X2; Sqh, myosin light polypeptide 9 isoform X1; Lyz1, putative lysozyme; Nurf-38, inorganic pyrophosphatase; SPE, serine protease easter-like; ApoLp-III, apolipophorin-III precursor; PPAE, prophenoloxidase activating enzyme precursor; CYP6B2, cytochrome P450; PGRP-LB, peptidoglycan-recognition protein LB-like; akr2e, aldo-keto reductase AKR2E4-like; Chit1, Glycosyl hydrolases family 18; ACLY, ATP-citrate synthase-like; MASP1, serine protease gd-like; ACD, acyl-CoA desaturase-like; GAPDH, glyceraldehyde-3-phosphate dehydrogenase.

To determine the reliability of the transcript data, 14 DEGs related to innate immunity, detoxification, and metabolism were selected upon performing real-time PCR using the glyceraldehyde-3-phosphate dehydrogenase (*GAPDH*) gene for normalization. As shown in **Figure 8**, the relative expression of qRT-PCR exhibited good consistency with the log_2_FC of the RNA-seq data, which indicated that our findings using RNA-seq were reliable and credible.

## Discussion

### PS can enhance host immunity by regulating the expression of genes related to immune signal recognition and prophenoloxidase activation

To reveal the mechanisms underlying the immune regulation on silkworm by PS, the GO terms and KEGG pathways corresponding to DEGs in the control *vs*. PS were analyzed. According to the results, a lot of DEGs were functionally annotated as “immune system process”, “response to stimulus”, and “metabolic process”. Meanwhile, many DEGs were annotated as “metabolism” category, including “carbohydrate metabolism”, “lipid metabolism”, and “amino acid metabolism” subcategories, in the KEGG database. Our finding indicated that PS could significantly enhance the immune system and altera the physiology metabolism of silkworm. Our results are consistent with previous transcriptome studies, which showed that *Atractylodis macrocephalae* Koidz polysaccharides exert its immunoregulation in lymphocyte by modulating the immune signaling pathway (23) and that dietary *Astragalus* polysaccharides could positively influence the innate immune response of grass carp (24).

It is known that the immune system of insects consists of humoral and cellular immunity. Induction of the Toll or Imd pathway by pathogen-associated molecular patterns (PAMPs) leads to the activation of humoral immunity and synthesis of certain AMPs. A total of 11 putative Toll-related receptors and two Toll analogs have been identified in the silkworm genome by Chen et al. (25), and they speculated that these receptors may play a key role in immune defense or other biological functions. Our transcriptome data showed that genes encoding pattern recognition receptor (PRR), including Toll and scavenger receptor (Scarb1 and SCARB2), were significantly increased in the fat body of silkworm after injection with PS. Scavenger receptor plays a critical role in activating hemocyte phagocytosis and inducing the expression of AMPs (26–28), so the upregulation of *Scarb1* and *SCARB2* genes induced by PS revealed a positive significance in antimicrobial, antiviral, and phagocytic functions for the host. The significant increases in the phagocytic activity of hemocytes in the silkworm after PS injection support this opinion. Moreover, similar results were found in RAW264.7 cells, such that polysaccharides can exert their immunomodulatory effect by enhancing the phagocytic activity (29). It is well known that *Spaetzle* is a key gene in the Toll pathway. Spaetzle protein can activate the Toll receptor and recruit the downstream protein, including MyD88, Tube, and the kinase Pell, to form a complex, which initiates downstream signal cascades (30). In this study, after PS exposure, the transcriptional levels of Toll and Imd signaling pathway genes, such as *Toll*, *Imd*, *Myd88*, and *Spaetzle*, were significantly upregulated, which was consistent with the upregulation of *Cecropin D* and *gloverin 1* genes. These results suggested that PS can activate AMP expression through the Toll or Imd signal pathway in silkworms. Noticeably, numerous research have also demonstrated that polysaccharides have the function of regulating host immunity and activating the immune cells effectively in vertebrates. They not only can act directly on natural killer cells, dendritic cells, and macrophages but also effectively activate T/B lymphocytes or regulate the secretion of cell cytokines (31, 32). However, a more detailed experiment is needed in the future to elucidate the underlying mechanism of the signal transduction pathway triggered by PS in silkworm.

We detected a high transcriptional level of ryanodine receptor genes in the fat body of PS injection silkworm, which have been demonstrated to be involved in the cellular immune defenses of insects, such as phagocytosis, hemocyte spreading, and encapsulation (33, 34). Our results suggested that PS can regulate the cellular immunity of silkworm, and they were further confirmed by the significant increases in hemocyte spreading ability of the silkworm after PS treatment. Differential gene expression analysis also showed that many genes related to the protophenol oxidase system were upregulated, such as *PPAE*, *proPO*, *MASP1* and *SPE*, *lint*, *Spn42Dd*, and *SPS-like*. These genes are the members of a proPO cascade, which has been demonstrated to be one of the key immune defense mechanisms in silkworms (35). In this cascade, proPO is first cleaved by PPAE, which also exists in silkworm tissue in the form of inactive zymogen (pro-PPAE). The proteolytic processing both of pro-PPAE and proPO leads to the formation of a large number of immune effector molecules such as AMPs, opsonic, and agglutinin and to the promotion of hemocyte differentiation (36). A previous study reported that *PPAE* transcripts were expressed in the hemocytes, integument, and salivary glands but not in the mid-gut or fat body of silkworm (37). Another study showed that the transcript abundance of *SPE* in *Anopheles gambiae* increased significantly following a bacterial infection (38). It has also been demonstrated that serine protease inhibitor (serpin), such as *Spn28D* in *D. melanogaster* and *Spn 40*, *Spn 55*, and *Spn 48* in *Tenebrio molitor*, revealed upregulated transcript levels and participated in modulating the melanization reaction during a pathogenic infection (39, 40). Combined with the results of the above-mentioned studies and our RNA-seq data, we can conclude that PS can trigger the proPO cascade and then convert proPO to the active form of phenoloxidase which is crucial for the formation of melanin and in the killing of invading pathogens. In fact, the significant increase of the PO activity detected in the fat body of PS-inoculated silkworm further confirmed the above-mentioned conclusion.

ApoLp-III acts as a PRR in insect and regulates the humoral and cellular immune responses, such as the synthesis of AMPs and immune-related proteins, nodule formation, and opsonization (41). It has been reported that the upregulation of BmApoLp-III in silkworm is beneficial for fighting against *Beauveria bassiana* (42). BmApoLp-III protein may be involved in the activation of the Toll pathway to enhance the defense ability of the silkworm against invaders. The synergistic action of lysozyme and apoLp-III against selected Gram-negative and Gram-positive bacteria was documented in *G. mellonella* larvae (43). In silkworm, we found that both lysozyme and apoLp-III were similarly upregulated, indicating that the host immune defenses were positively regulated by exogenous PS. In addition, we also observed that a significant decrease in PGRP-LB mRNA occurred in the fat body of PS treatment larvae. These results further confirmed that PGRP-LB played an important negative regulatory role in systemic immunity and intestinal epithelial immunity (44).

The GSEA showed that the PS treatment larvae exhibited highly enriched signatures for many immune signaling pathways, such as MAPK, PI3K-Akt, and peroxisome pathway compared with the control. It is well known that the MAPK signaling pathways, including p38, JNK, and ERK, functions in triggering host innate immune responses. Polysaccharide can activate the MAPK and NF-κB signal through triggering specific receptors, including TLR-4, scavenger receptor, complement receptor 3, and Dectin-1, and induce the expression of inflammation factors in macrophages (45). In humans, the MAPK can regulate the production of molecules such as IL-6, TNF-α, and nitric oxide (NO), which play an important role in immune response and inflammation (46). Furthermore, a previous study revealed that activating the MAPK signaling cascade is necessary for diamondback moth, *Plutella xylostella*, to overcome the toxic action of *Bacillus thuringiensis* (47), and there is also evidence to suggest that activation of MAPK signaling in *Drosophila* has contributed to restricting the strength of IMD signaling, which could prevent the occurrence of an excessive immune response (48). When insects initiate the immune system to fight against invaders, a huge number of reactive oxygen species (ROS) will be generated by the immune cells. An appropriate amount of ROS is conducive to the killing of invaders, but excessive levels of ROS are toxic. The peroxisome pathway is involved in the detoxification of ROS and also mediate the cytoskeletal rearrangement required for phagocytosis (49). The PI3K-Akt pathway plays an essential role in several biological processes such as cell apoptosis, proliferation, and differentiation (50). To summarize, the GSEA in this study displayed an overview of various regulated genes, suggesting that PS are capable of regulating the immune functions of silkworm through activating the MAPK, PI3K-Akt, and peroxisome signaling pathways.

### Metabolic changes in the fat body of silkworm after inoculation with *Poria cocos* polysaccharides

In this study, we found that many DEGs participated in carbohydrate metabolism (*e*.*g*., pentose and glucuronate interconversions, ascorbate and aldarate metabolism, galactose metabolism, and amino sugar and nucleotide sugar metabolism) and lipid metabolism (*e*.*g*., steroid hormone biosynthesis, glycerolipid metabolism, and fatty acid biosynthesis). Most of the DEGs in these pathways were upregulated in the experimental group, such as *pik3c3*, *UGT2A3*, *CRYL1*, *Chit1*, *Elovl4*, *EGT*, *FAS*, *TE*-domain, and *pksM*. These results indicated that PS injection enhanced the level of carbohydrate and lipid metabolism in the fat body of silkworm. The increased enzyme activities, including G6PD, HK, and FAS, related to carbohydrate metabolism or lipid metabolism in the fat body of PS treatment silkworm further confirmed the above-mentioned opinion. G6PD is an important enzyme for NADPH biosynthesis, and a high level of G6PD activity could extend the lifespan of transgenic *Drosophila melanogaster* by improving the oxidative stress (51). HK is a key enzyme that controls glycolysis because it can phosphorylate glucose, fructose, and mannose to the corresponding hexose 6-phosphates (52). It also acts as a PRR for the recognition of various PAMPs and activation of an anti-bacterial immune response (53). FAS catalyzes acetyl coenzyme A (acetyl-CoA) and malonyl-CoA to produce palmitate, which plays important roles in adenosine triphosphate (ATP) production (54). Large amounts of ATP are needed for insects to activate their innate immune responses.

The KEGG-based GSEA also revealed that glycolysis, TCA cycle, glycerolipid metabolism, and fatty acid biosynthesis were significantly enriched gene sets, with higher expression levels in the PS treatment group compared with the control group. These changes enabled the immune cells to respond effectively during a pathogen infection. In mammals, this similar phenomenon is called Warburg effect (55). The TCA cycle is the central metabolic hub of sugars, lipids, and amino acids and finally produces a large amount of ATP through oxidative phosphorylation (56). As we all know, the insect fat body is a key nutrient metabolic organ which plays an important role in storing energy and providing energy to the rest of the organism, but it is also an organ important for exerting immune responses. The upregulated transcriptions of genes involved in Toll or Imd signal pathway and the increase in cellular immune parameters in silkworm after a PS injection indicated that PS could activate the innate immune responses of silkworm. However, the activation of the immune system leads to a high level of energy consumption (57); therefore, the organism will undergo a metabolic switch or metabolic reprogramming, which supplies adequate energy to synthesize immune effector molecules. Metabolic reprogramming, including glucose consumption or glycolysis, has been well documented in mammalian cells after immune stimulation (57). However, such changes in energy metabolism have received little attention in insects. It has been demonstrated that mounting humoral (synthesis of AMPs and activation of proPO system) and cellular (phagocytosis, encapsulation, and nodulation) immune responses are energy-consuming processes; the energy requirements of immune cells may rise from 10% to almost 30% of the whole energy consumption (58). The increase in energy consumption and the rapid production of new biomolecules lead to the metabolic conversion of immune cells. In other words, carbohydrate and lipid metabolism must be synchronized with these changes in energy requirements. The mRNA levels of genes encoding ACLY, akr2e, and Aldh1b1, which participate in ATP synthesis, were also significantly increased, further indicating that energy metabolism and ATP production were enhanced in the PS-treated silkworm. Our results suggested that PS can enhance the energy metabolism of *B. mori* larvae by activating carbohydrate and lipid metabolism, especially glycolysis and TCA cycle, so as to meet the needs of high-intensity immune ability. This is supported by a previous study which examined an increase in ATP production due to the energy requirements of mounting an immune response in LPS-challenged *Gambusia holbrooki* (59). PS could also rescue the acetaminophen-induced liver injury by suppressing the excessive inflammatory response and apoptosis (60).

The COG annotation analysis revealed that a lot of DEGs were annotated to amino acid transport and metabolism. Many DEGs involved in amino acid metabolism, such as *Gnmt*, *HPD*, *Chsy1*, *DNMT1*, and *FAH*, were upregulated in the PS treatment group. Amino acids and their metabolites participate in the activation of cellular immunity, including cell proliferation, differentiation, phagocytosis, nodulation, and encapsulation, as well as in the synthesis of immune-related molecules and regulation of immune signaling pathways and oxidative stress—for instance, arginine (Arg) can be converted into nitric oxide NO, which functions as a regulator of many immune cells (61). Dietary Trp improved the hemocyte phagocytic activity and increased the total hemocyte count, hemocyanin, acid phosphatase, and alkaline phosphatase activity of *Eriocheir sinensis* (62). Therefore, combined with our results, we can conclude that PS may improve the immune system by regulating the amino acid metabolism.

## Conclusions

The transcriptome data revealed that many DEGs involved in energy metabolism, including carbohydrate metabolism, lipid metabolism, and amino acid metabolism, were significantly upregulated in the fat body of silkworm after an injection with PS. The gene expression profiles, innate immune parameters, and enzyme activity assays demonstrated that PS could enhance the immunity of silkworm by increasing hemocyte phagocytosis, microaggregation, and spreading ability as well as upregulating the expression levels of genes related to immune signal recognition, detoxification, and proPO activation. These findings provided us valuable insights into how PS regulated the host immune and physiology systems and broadened our understanding of the application of polysaccharides as drugs and vaccine adjuvant.

## Data availability statement

The data presented in the study are deposited in the SRA repository, accession numbers SRR18297772, SRR18297773, SRR18297774, SRR18297778, SRR18297779, and SRR18297780.

## Author contributions

Wrote the paper: GW. Performed the experiments: JL and WH. Provided suggestions for the experiments and manuscript: YX, ML and YL. Designed the experiments: YY and GW.

## Funding

This study was supported by the National Natural Science Foundation of China (31701852, 31802135), Characteristic Innovation Projects of Colleges and Universities in Guangdong Province in 2019 (2019KTSCX074), Major Scientific Research Projects of Social Public Welfare in Zhongshan City (2019B2013), Engineering Technology Research Center of Gene and Bioinformatics (Zhongshan Institute) in Zhongshan City (170702100193188), and Gene and Protein Engineering Technology Research Center of Guangdong Province (418S43).

## Conflict of interest

The authors declare that the research was conducted in the absence of any commercial or financial relationships that could be construed as a potential conflict of interest.

## Publisher’s note

All claims expressed in this article are solely those of the authors and do not necessarily represent those of their affiliated organizations, or those of the publisher, the editors and the reviewers. Any product that may be evaluated in this article, or claim that may be made by its manufacturer, is not guaranteed or endorsed by the publisher.

## Supplementary material

Supplementary Table 1 List of immune and metabolism-related DEGs primers used for qPCR validation.

Supplementary Table 2 Statistical analysis and quality evaluation of RNA-seq data.

Supplementary Figure 1 Quality assessment of the transcriptome data. **(A)** Pie chart of reads distribution in different genomic regions. **(B)** Violin plot of the expression distribution of different samples. The bulky part of each sample represents the region with the most concentrated gene expression in the whole sample, the black rectangle encompasses the lower and upper quartiles, and the white dot represents the median. **(C)** The reads distribution in chromosomes for different samples. The abscissa represents different chromosomes; the ordinate represents the number of reads that mapped to the corresponding chromosome.

Supplementary Figure 2 Gene Set Enrichment Analysis (GSEA) enrichment plot of significantly enriched pathways related to immune response and metabolism as identified by GSEA-based Kyoto Encyclopedia of Genes and Genomes analysis. **(A)** MAPK signaling pathway, **(B)** peroxisome, **(C)** PI3K-Akt signaling pathway, **(D)** fructose and mannose metabolism, **(E)** galactose metabolism, **(F)** citrate cycle (TCA cycle), **(G)** glycolysis/gluconeogenesis, **(H)** fatty acid biosynthesis, and **(I)** glycerolipid metabolism.
