# Supplementary figures and images for: Transcriptome analysis reveals immune and metabolic regulation effects of Poria cocos polysaccharides on Bombyx mori larvae
Source: Front Immunol. 2022 Oct 28;13:1014985. doi: 10.3389/fimmu.2022.1014985 (PMC9650554; doi:10.3389/fimmu.2022.1014985)

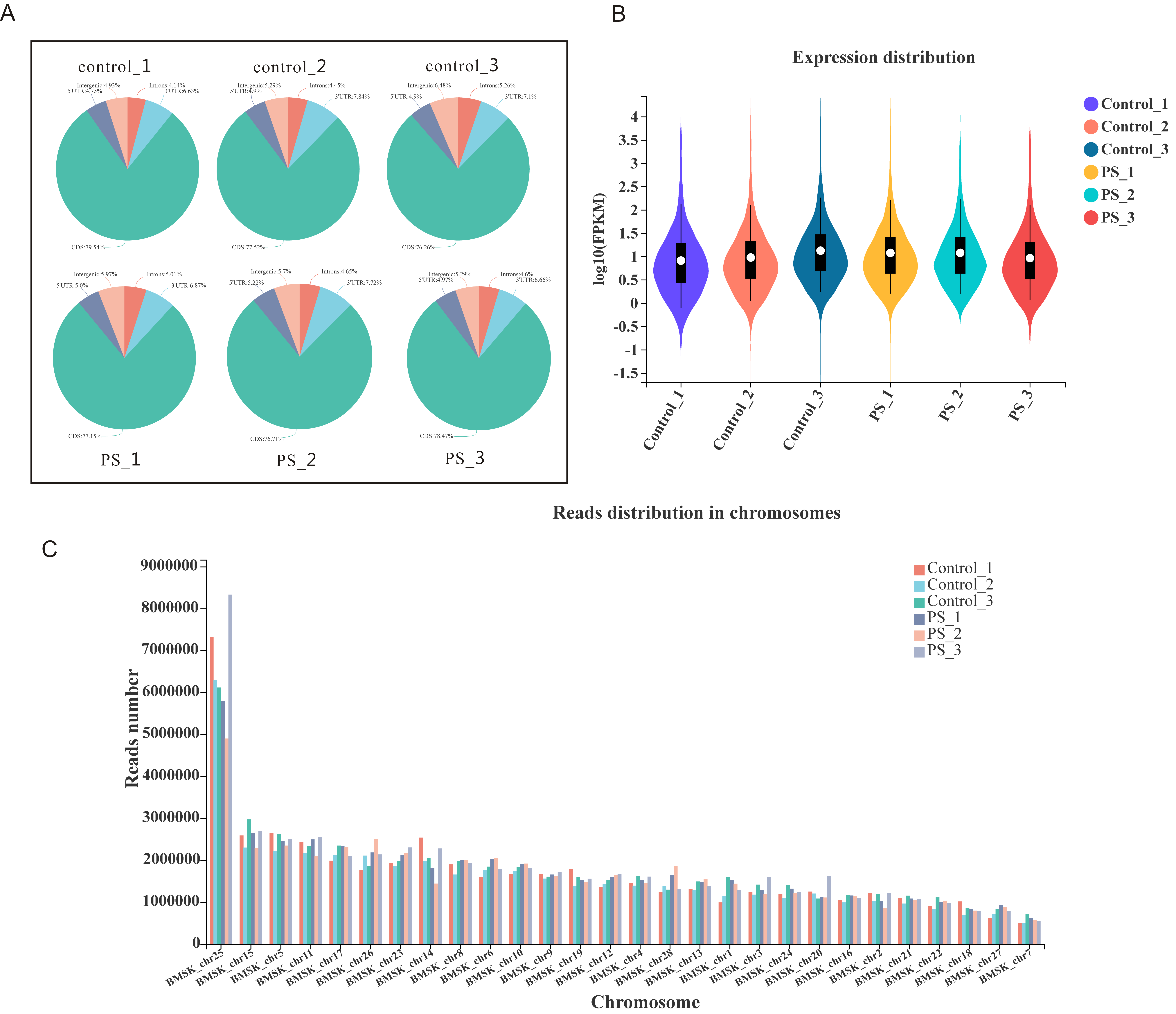

Supplement: Supplementary Figure 1 — Quality assessment of the transcriptome data. (A) Pie chart of reads distribution in different genomic regions. (B) Violin plot of the expression distribution of different samples. The bulky part of each sample represents the region with the most concentrated gene expression in the whole sample, the black rectangle encompasses the lower and upper quartiles, and the white dot represents the median. (C) The reads distribution in chromosomes for different samples. The abscissa represents different chromosomes; the ordinate represents the number of reads that mapped to the corresponding chromosome. [file Image_1.tif]

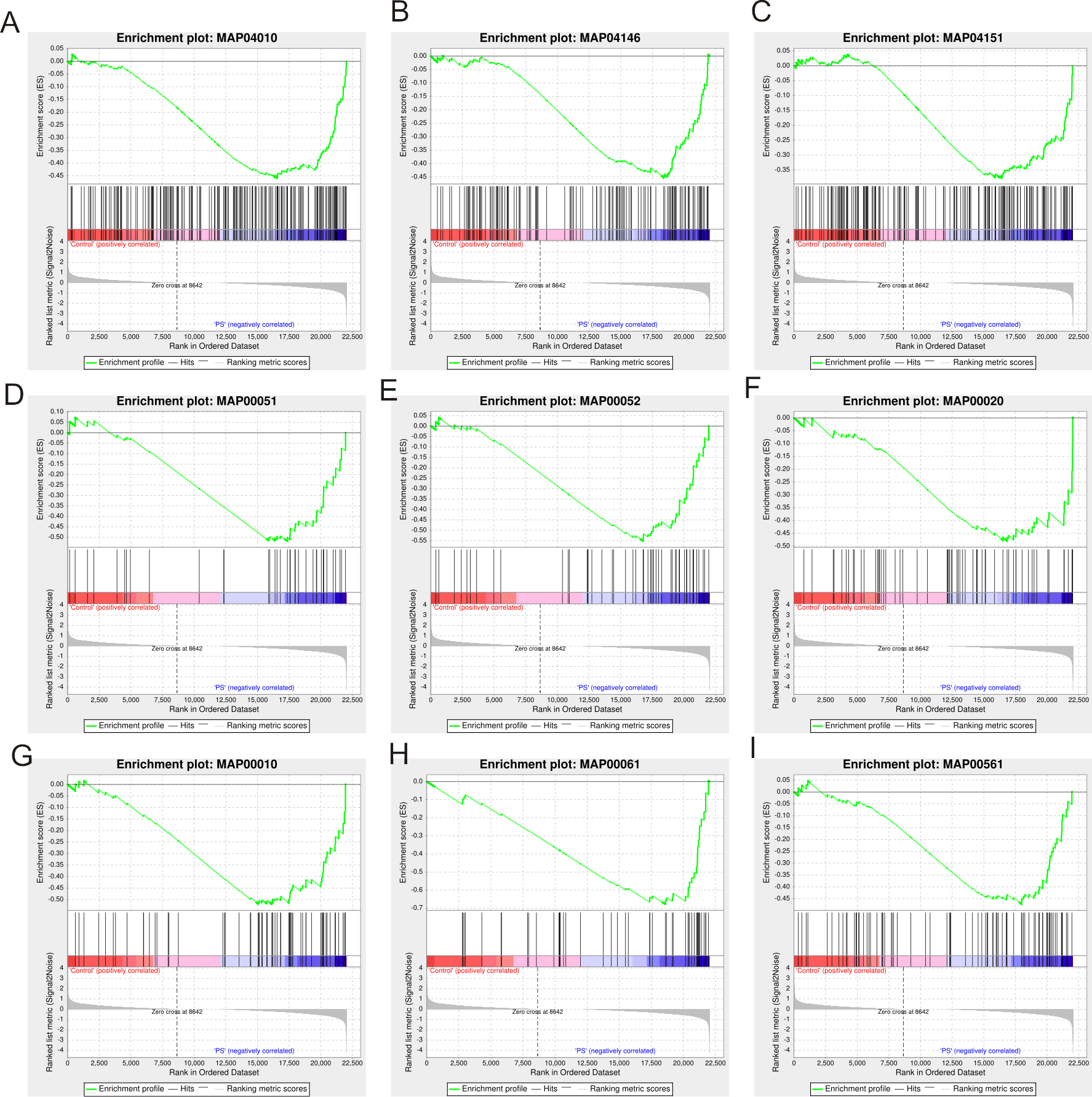

Supplement: Supplementary Figure 2 — Gene Set Enrichment Analysis (GSEA) enrichment plot of significantly enriched pathways related to immune response and metabolism as identified by GSEA-based Kyoto Encyclopedia of Genes and Genomes analysis. (A) MAPK signaling pathway, (B) peroxisome, (C) PI3K-Akt signaling pathway, (D) fructose and mannose metabolism, (E) galactose metabolism, (F) citrate cycle (TCA cycle), (G) glycolysis/gluconeogenesis, (H) fatty acid biosynthesis, and (I) glycerolipid metabolism. [file Image_2.tif]
